# Utilization of Iron Tailings Sand as an Environmentally Friendly Alternative to Natural River Sand in High-Strength Concrete: Shrinkage Characterization and Mitigation Strategies

**DOI:** 10.3390/ma13245614

**Published:** 2020-12-09

**Authors:** Zhiqiang Zhang, Zhilu Zhang, Shaoning Yin, Linwen Yu

**Affiliations:** 1 College of Materials Science and Engineering, Chongqing University, Chongqing 400045, China; zzqiang1962@126.com; 2 Changzhou Green Mart Constrcution Technology Co., Ltd., Changzhou 213015, China; 20160902038@cqu.edu.cn; 3 Technical Supervision and Research Center of the Building Materials Industry, Beijing 100024, China; 20160913136@cqu.edu.cn

**Keywords:** iron tailings sand, concrete, shrinkage, mitigation strategies

## Abstract

The increasing annual emissions of iron ore tailings have proved a great threat to the natural environment, and the shortage of natural river sand, as well as the pursuit of sustainable development materials, provides motivation to reuse iron ore tailings as a fine aggregate in concrete. Due to the significantly different properties of iron tailings sand compared with natural river sand—such as the higher density, higher content of limestone particles smaller than 75 μm and its rough and angular shape—concretes prepared with iron tailings sand show remarkably higher shrinkage. This study presents the shrinkage characterization and shrinkage-reducing efficiency of three different methods on iron tailings, sand concrete and river sand concrete. The internal humidity was also monitored to reveal the shrinkage-reducing mechanism. The obtained results indicated that the autogenous and total shrinkage of iron tailings sand concrete were 9.8% and 13.3% higher than the river sand concrete at the age of 90 d, respectively. The shrinkage reducing agent (SRA) was the most effective shrinkage reducing method for river sand concrete, while for iron tailings sand concrete, super absorbent polymer (SAP) and controlled permeable formwork liner (CPFL) it worked best on autogenous shrinkage and drying shrinkage, respectively. Furthermore, the shrinkage mitigation strategies worked earlier for the drying shrinkage behavior of iron tailings sand concrete, while no such condition could be found for autogenous shrinkage.

## 1. Introduction

In recent years, the worldwide mining of ferrous and other metallic ores has been increasing to answer the ever-growing need for various alloys used in daily life. With the rapid development of iron and steel industry in recent years all around the world, iron ore tailings, which result from ore dressing, account for an increasing proportion of industrial solid waste. It is estimated that for each tonne of beneficiated iron ore, 400 kg of tailings are produced [1]. It was estimated that about 632 million tonnes of iron ore tailings are generated yearly in western Australia, and more than 275 tonnes in Brazil [1,2]. According to an official report, the stockpiles of iron ore tailings increased from 536 to 839 million tons during a period of five years since 2009, and this number still continues to increase year by year. However, only less than 10% of the iron ore tailings had been recycled as resources. The storage and landfill of remaining untreated tailings occupy a large amount of soil. It would also make the soil lack organic carbon and basic nutrients, which would be unsuitable for the establishment and colonization of plants [3]. The storage of iron ore tailings needs to build tailing ponds at the capital outlay of 1–3 Yuan per 1 t, and a management fee of 3–5 Yuan per 1 t [4], resulting in total costs of disposing tailings more than 70 million Yuan per year in China. Furthermore, the construction of tailings ponds could also give rise to security risks, such as tailings dam-break accidents [5,6]. Meanwhile, toxic and harmful substances in tailings (such as heavy metal ions and other harmful chemicals) would bring serious pollution to the environment [5,7]. On the other hand, due to the destruction of river channels during the sand mining process and the increasing awareness of environmental protection in China, river sand mining has been restricted. Therefore, the existing natural river sand resources cannot meet the needs of massive engineering construction. One way of disposing of these iron ore tailings is to utilize in-concrete production after the crushing and screening process [4,6], which could also alleviate the shortage of river sand [8].

Recent researches have shown that iron tailings sand has potential to produce concrete by replacing river sand. Ugama et al. [9] investigated the properties of a rigid pavement using iron tailings sand, and their study indicated that the workability, compressive strength and indirect tensile strength decreased with increasing tailings sand replacement ratio. Zhao et al. [7] reported that 100% replacement of natural aggregate with iron tailings sand in ultra-high-performance concrete resulted in a reduced flow value and mechanical strength, but no significant impact could be found when the tailings sand content was lower than 40%. Liu et al. [10] evaluated the feasibility of utilizing iron tailings sand preparing sprayed concrete in terms of mechanical performances. Their results showed that 20% replacement of river sand with tailings sand was best. Both Wang [11] and Ma et al. [12] developed a new type of autoclaved aerated concrete with iron tailings sand, and the properties of their concretes achieved the requirements of Chinese standard GB/T 11969-2008. 

The application of iron tailings sand in normal or high-performance concrete was also investigated. Zhang et al. [13] investigated the properties of high-performance concrete prepared by a mix of iron tailings sand and manufactured sand. They found that the compressive strength of concrete showed downtrend with increasing iron tailings sand content, but the frost resistance of concrete was comparable with pure river sand concrete. Kuranchie et al. [2] reported that 11.6% of compressive strength enhancement and 16% of split tensile strength reduction were obtained for iron tailings sand concrete, compared with conventional aggregates concrete. The resistance to corrosion and acid attacks was also improved by using tailings sand. However, this result differs from the observations of Shettima et al. [14], who reported that higher split tensile strength, as well as compressive strength and elasticity modulus of concrete with iron tailings sand, was found. They also showed that the carbonation depth and drying shrinkage of tailings sand concrete were lower than river sand concrete, while the water absorption rate and chloride penetration increased with tailings sand proportions. 

The majority of research on iron tailings sand for concrete infrastructures focuses on mechanical and durability performance, while experimental studies on shrinkage properties are rarely found. However, shrinkage properties are vital to the service life of concrete structures, since high shrinkage may induce cracks, which would intensify the intrusion of aggressive substances [15,16,17]. In this respect, the shrinkage characteristics of concrete with river sand and iron tailings sand were investigated, along with general quality control properties including slump, slump flow, compressive and flexural strength and dynamic elasticity modulus. Our preliminary test results showed that the shrinkage of iron tailings sand concrete was significantly higher than that of river sand concrete. Adding a superabsorbent polymer (SAP) and a shrinkage reducing agent (SRA) is a common method to mitigate the shrinkage of concrete [18,19,20]. Furthermore, controlled permeable formwork liner (CPFL) is also an effective technology that improves the quality of cover concrete [21,22]. It has been confirmed that CPFL covered on the surface of concrete mitigated early age shrinkage effectively, due to the improved internal relative moisture content of surface concrete [23]. In this study, we focused on the impacts of several shrinkage-reducing measures, including adding SAP, SRA and applying CPF liner, on the shrinkage performance of concrete containing iron tailing sand.

## 2. Materials and Methods 

### 2.1. Raw Materials

#### 2.1.1. Cement and Aggregate

Portland cement with a Blaine fineness of 350 m^2^/kg, P·O 42.5, confirming to Chinese standard GB175-2007 was obtained from Chongqing Fuhuang company. Ground granulated blast furnace slag (GGBFS) with a specific surface area of 430 m^2^/kg was also provided by this company. Fly ash was supplied by the Chongqing Luohuang power plant. The chemical composition of cement, GGBFS and fly ash were characterized by the X-ray fluorescence (XRF) method, and is shown in Table 1. 

**Figure 1:**
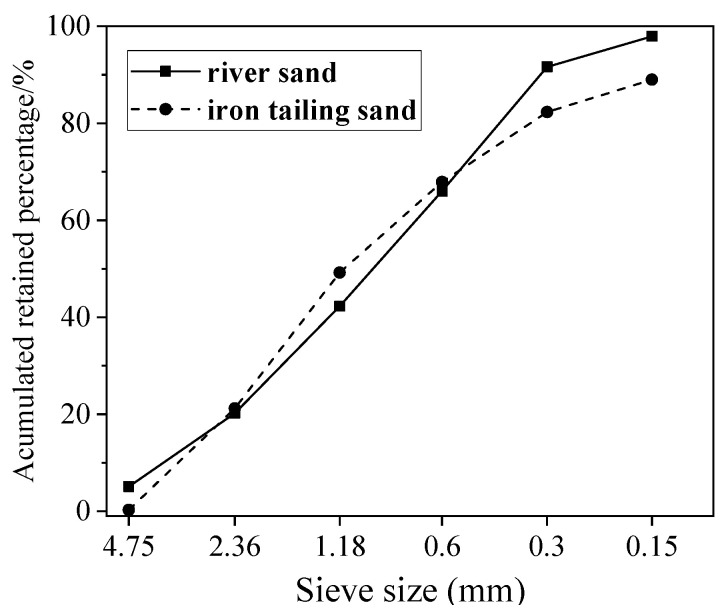
Sieve analysis of iron tailings sand and river sand.

The coarse aggregate used was crushed limestone with two grades (5–10 mm and 10–20 mm), mixed with a ratio of 4:6 by mass. One of the fine aggregates was river sand with fineness modulus of 3.10, and its sieving curve is reported in Figure 1. Its bulk density was 2.68 g/cm^3^.

#### 2.1.2. Iron Tailings Sand

**Figure 2:**
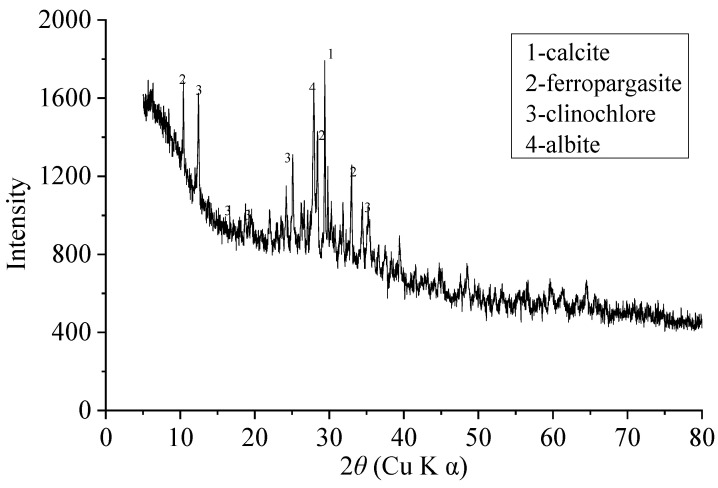
Mineral composition of iron tailings sand.

**Figure 3:**
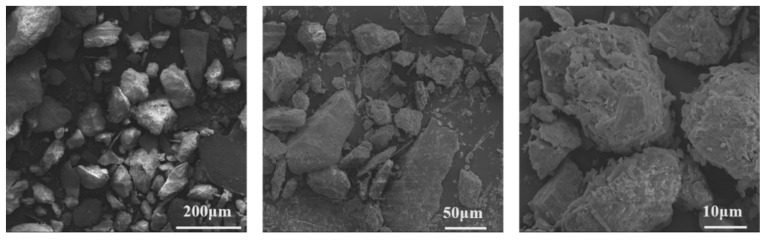
SEM images of iron tailings sand.

Iron tailings sand was provided by a mining group in Shangluo, Shanxi province. The bulk density and fineness modulus of iron tailings sand were 3.03 g/cm^3^ and 3.10, respectively. Its sieving curve is presented in Figure 1. The iron tailings sand contained 5.6% of powders smaller than 75 μm, and its chemical composition, characterized by the XRF method, is reported in Table 2. It is clear that the tailings sand had a Fe_2_O_3_ content as high as 16.96%, which resulted in a significantly higher bulk density than river sand. The X-ray diffraction (XRD) spectrograms (Rigaku, Austin, TX, USA) of the iron tailings sand in Figure 2 show the presence of calcite, ferropargasite, clinochl and albite. Figure 3 shows the SEM images of iron tailings sand at different magnifications. The SEM (TESCAN, Kohoutovice, Czech Republic) analysis showed that the shape of tailings sand was rough and angular.

#### 2.1.3. Shrinkage Reducing Methods

**Figure 4:**
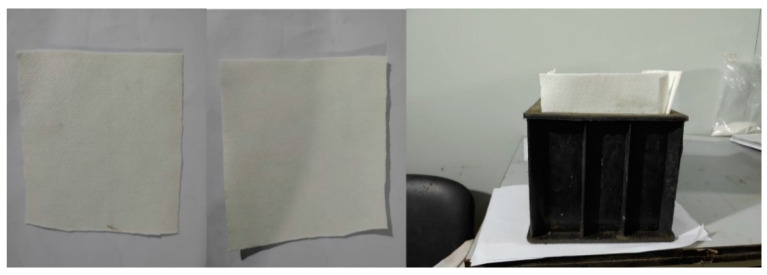
Appearance of the controlled permeable formwork liner.

The SAP (Xinya, Changzhou, China) was a commercial product supplied by a materials company in Jiangsu province. The main composition was cross-linked sodium polyacrylate [-CH_2_-CH(CO_2_Na)]. All the particles passed the sieve of 200 mesh. Each gram of SAP can absorb about 300 mL of water. The SRA was obtained from Sobute New Materials company (Jiangsu, China), and its recommended dosage is 0.6~1.2% (percent mass of the binder content). The CPFL used in this study was provided by Huimin, Shengzhen, China. The appearance and properties of CPFL were shown in Figure 4 and Table 3, respectively. The CPFL was pasted on four sides of the molds by coating the glue evenly, and removed from the concrete surface after a time of 3 d.

### 2.2. Mix Proportions

Table 4 provides mix proportions of concrete incorporating iron tailings sand. Studies [7,11,12,13] have shown that iron tailings sand could be used in concrete as sand replacement, and in order to maximize the effect of iron tailings sand in reducing environmental problems, cost and natural resources depletion, a 100% replacement ratio was used in this study. A polycarboxylate superplasticizer of 1% was used as admixture in all the concrete mixtures. SAP and SRA were added as shrinkage mitigation methods, as well as the application of CPFL. It should be mentioned that due to the high water-absorption ability of SAP, the workability of concrete would decrease severely if the same water to binder ratio was used. Therefore, in order to maintain the same workability of SAP containing concretes with other mixtures, additional water, which was marked as Wa/B, was determined through the “tea-bag” method [24] by absorbing a filtrated cement paste solution. The addition of SRA did not significantly affect the workability. Finally, all the concrete had a slump ranging between 190–200 mm.

### 2.3. Test Methods

#### 2.3.1. Mechanical Properties

The compressive and flexural strength of concrete were tested at ages of 7 d and 28 d, and the dynamic modulus of the elasticity of concrete was also measured at 28 d. The samples for the compressive strength test are cubes with a dimension of 100 mm × 100 mm × 100 mm. All those results were the average of three concrete samples.

#### 2.3.2. Drying and Autogenous Shrinkage

The drying shrinkage of concrete was determined according to Chinese standard GB/T 50082-2009. Iron tailings sand and river sand concrete was cast in 100 mm × 100 mm × 515 mm steel molds and vibrated on a vibrating table until no air bubbles appeared on the surface, followed by covering with plastic film to avoid water evaporation. The concrete specimens were cured in a room with a temperature of 20 ± 2 °C and a relative humidity (RH) of 95%. After 1 d, concrete samples were demolded and subsequently cured in the same environment for another 2 d. Then, the specimens were transferred into a room with a temperature of 20 ± 3 °C and an RH of 60 ± 5%. The length changes along the longitudinal axis were measured immediately by a dial gauge.

The autogenous shrinkage followed the same procedure with the drying shrinkage test, while concrete samples were sealed by Teflon film after 3 d of standard curing, and the initial length was also recorded after the specimens were transferred into a room with a temperature of 20 ± 3°C. The shrinkage value of autogenous and drying shrinkage of concrete were the average of three samples.

#### 2.3.3. Internal Relative Humidity

**Figure 5:**
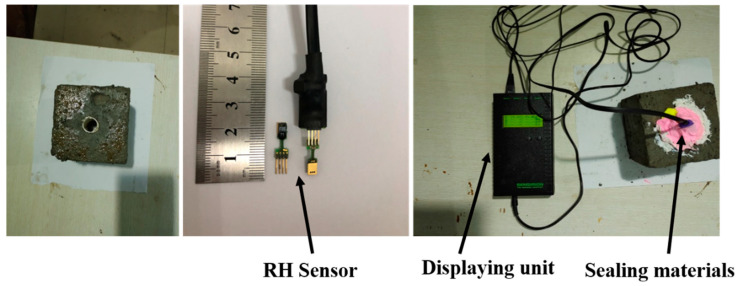
Internal relative humidity (RH) test setup.

An internal RH test was carried out on 100 mm cubic samples by using a humidity sensor (Figure 5). Before casting, cylindrical plastic pipes with a diameter of 15 mm and a height of 20 mm were fixed to the center of steel molds, and the inside of pipes were filled with plasticine to prevent the injection of cement paste. After casting, the plasticine was removed and pipes were sealed with rubber plugs. Silicone was also used to seal the gaps between plugs and pipes. Before the measurements, the calibration of the humidity sensor was carried out with four saturated salt solutions (KNO_3_, KCl, NaCl and Mg(NO_3_)_2_) with a known constant RH in the range of 55–100% RH. The accuracy of the sensors was within ±1.8% RH. The average of two replicate measurements was reported.

#### 2.3.4. Chloride Penetration Test

Rapid chloride penetration tests were carried out according to ASTM C1202. Cylindrical samples with a diameter of 100 ± 1 mm and a height of 50 ± 2 mm were used to determine the resistance to chloride penetration by total charge passed through method at the age of 28 d.

## 3. Results and Discussion

### 3.1. General Properties

Table 5 summarizes the general properties of concrete prepared by river sand and iron tailings sand. It is clear that replacing river sand with iron tailings sand did not make any significant impact on those performances. To be more specific, the slump of iron tailings sand concrete was a little lower than river sand concrete, while the compressive strength, flexural strength and elasticity modulus were slightly improved. The similar sieving curves and fineness modulus of the two sands resulted in the comparable slump. The slightly lower slump of iron tailings sand concrete could be ascribed to the angular shape of iron tailings sand. However, different results were reported by Shettima et al. [14], who found that the workability of concrete reduced with increasing iron tailings sand content. This could be attributed to the difference in the fineness modulus of iron tailings sand (3.1 in this study, 1.05 in Shettima’s work), since the decrease of fineness modulus would reduce the workability of concrete by increasing the total appearance area and water demand [25,26].

### 3.2. Autogenous and Total Shrinkage

**Figure 6:**
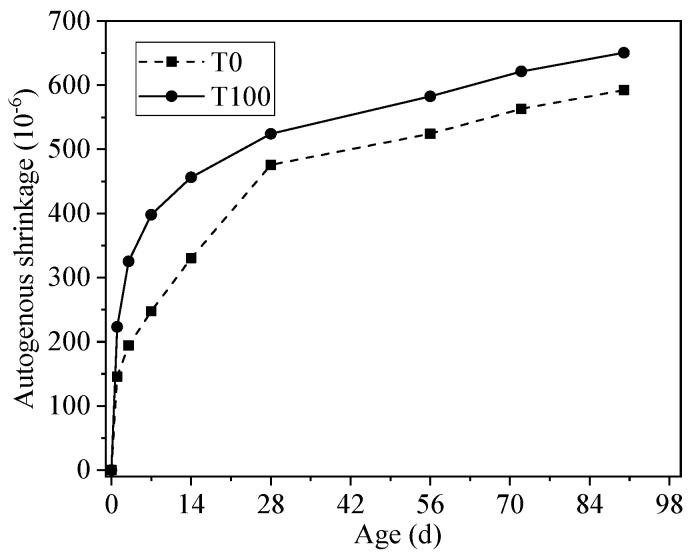
Autogenous shrinkage of concrete with iron tailings sand and river sand.

The autogenous shrinkage of concrete includes volume deformation caused by chemical shrinkage and self-desiccation [15,27]. The consumption of water in capillary pores due to the hydration of cement particles will cause capillary stresses, which is a main driving force for autogenous shrinkage and drying shrinkage [16]. Figure 6 shows the autogenous shrinkage of concrete with river sand and 100% iron tailings sand in 90 days. It is clear that iron tailings sand concrete presented higher shrinkage than river sand concrete during the entire test period, and its shrinkage value at 90 d was 9.8% higher than the control specimen. This is probably due to three reasons, with the first being that the density of the iron tailings sand (3.03 g/cm^3^) is larger than the river sand (2.68 g/cm^3^); it could be calculated that the volume ratio of fine aggregate in concrete decreased from 27.8% to 24.0%, when the river sand was replaced by the iron tailing sand of the same mass. Therefore, it results in a higher shrinkage for iron tailings sand concrete, since sands are dimensionally stable. The second reason is that the cement paste content was higher in concrete made by iron tailings sand due to its lower volume ratio, which could also account for the higher shrinkage deformation of iron tailings sand concrete. The third is a higher content of particles smaller than 75 μm, which was identified as calcite in the XRD spectrograms of the iron tailings sand. It was reported that limestone could provide a lower nucleation energy barrier compared with quartz during the nucleation process of C-S-H, and also provide additional nucleation sites, thus accelerating the hydration rate of cement [28,29,30]. The increasing reaction rate of cement particles would lead to a higher chemical shrinkage and larger self-desiccation shrinkage due to the faster consumption of water.

**Figure 7:**
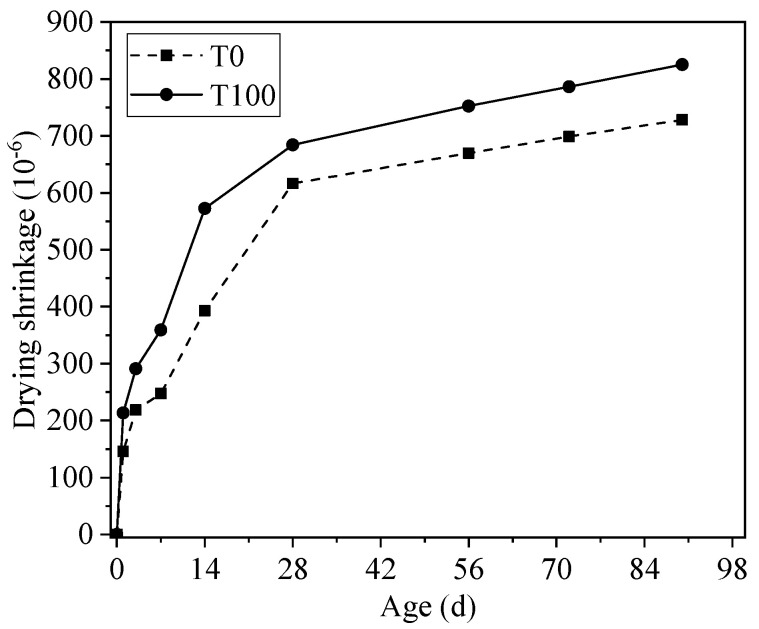
Total shrinkage of concrete with iron tailings sand and river sand.

Figure 7 illustrates the total shrinkage of iron tailings sand and river sand concretes in 90 days. The result shows that the replacement of river sand with iron tailings sand increased the total shrinkage of concrete by 13.3% at the age of 90 d. Considering the increase in the autogenous shrinkage, we can find the replacement of river sand by iron tailings sand resulted in a slight increase in the drying shrinkage. This result differs from the observations of Shettima et al. [14], who found that the drying shrinkage of concrete decreased continuously, with an increasing iron tailings sand replacement ratio. In this work, the author ascribed this result to the porous nature of iron tailings sand, which could absorb water and would release water during the drying process. However, no such structure could be identified in SEM observation in Figure 3. Apart from the reasons that resulted in a higher autogenous shrinkage of iron tailings sand concrete, another reason was that the shape of tailings sand was rough and angular, which resulted in a poor water-retaining property. Therefore, the moisture inside concrete would be removed easily and rapidly, leading to higher shrinkage.

### 3.3. Effects of Shrinkage Mitigation Methods on Shrinkage Properties of Concrete

**Figure 8:**
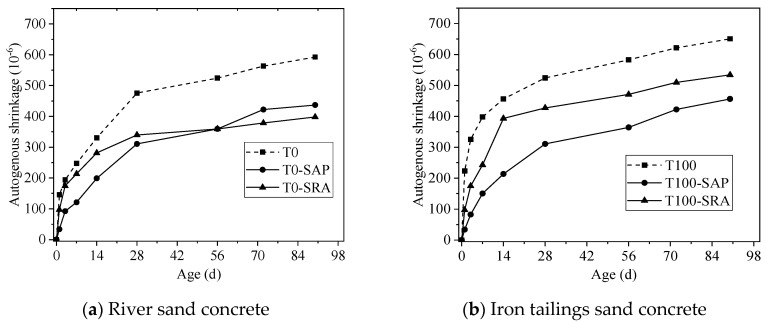
Effects of shrinkage mitigation methods on autogenous shrinkage of concrete.

The influence of SAP and SRA on the autogenous shrinkage of river sand concrete and iron tailings sand concrete are shown in Figure 8. As expected, the shrinkage of both types of concrete reduced significantly. The application of SAP and SRA could reduce the autogenous shrinkage of river sand concrete at 90 d by 26.2% and 32.8% respectively, while 29.9% and 17.9% reductions were observed in iron tailings sand concrete. It is clear that SRA performed more efficiently than SAP in river sand concrete, which was in line with the observations of Liu et al. [31]. However, the opposite trend was identified in iron tailings sand concrete, and a much lower reduction extent was also found when SRA was used. It is worth noting that the autogenous shrinkage of iron tailings sand concrete after using shrinkage mitigation methods was lower than river sand concrete, although it was still higher than river sand concrete using SAP and SRA.

**Figure 9:**
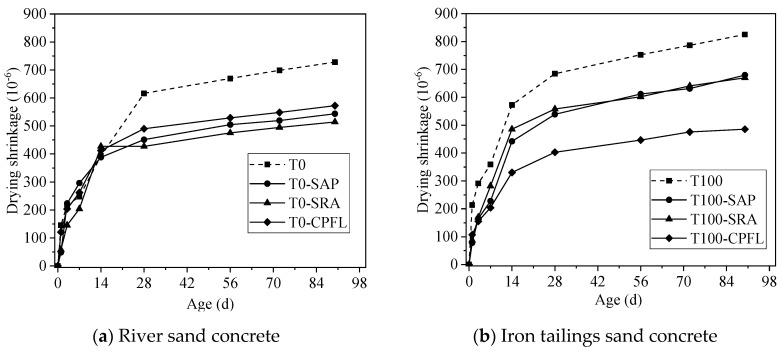
Effects of shrinkage mitigation methods on drying shrinkage of concrete.

The impact of shrinkage mitigation strategies on the total shrinkage of two types of concrete was presented in Figure 9. The results show that the use of SAP, SRA and CPFL had a remarkable reduction in the total shrinkage of concrete. For river sand concrete, SRA was still the most effective method to reduce shrinkage, where a 29.3% shrinkage decrease was observed by the addition of SRA and a lower reduction extent was identified with the use of SAP (25.3%) and CPFL (21.3%). However, no evident difference could be found in shrinkage reducing efficiency of SAP and SAR for iron tailings sand concrete. What’s more, it was the application of CPFL that prominently reduced the total shrinkage of concrete, achieving a 41.2% reduction rate. In addition, the use of shrinkage mitigation methods did not make a noticeable difference for the total shrinkage behavior of river sand concrete before 14 days, while much more obvious effects could be observed in iron tailings sand concrete after 3 days. It is worth mentioning that the total shrinkage of iron tailings sand after using shrinkage mitigation methods was lower than river sand concrete (control group).

The total shrinkage includes the autogenous shrinkage and the drying shrinkage. The reduce of total shrinkage caused by the application of SAP and SRA could mainly be ascribed to the decrease of autogenous shrinkage. SAP shows a superior desorption capacity, which can release water during cement hydration, such that this water can compensate water consumed by hydration, and thus increase the internal RH of the concrete and reduce the autogenous shrinkage [27,32]. The drying shrinkage of concrete in an air-dry curing condition was not significantly affected by the use of SAP, which has been confirmed by Kang et al. [33]. The SRA mainly reduces the shrinkage of concrete by decreasing the surface tension of the pore solution [34,35], although some studies [36] have reported that the SRA could also reduce the evaporation rate of water in the concrete. The application of CPFL can drain out air bubbles and water while retaining fine particles like cement, which would lead to a reduced water to binder ratio and the improved structure of the concrete surface [22,37,38]. Furthermore, water absorbed by the CPFL would be also released to compensate the RH drop of concrete cover [23]. All of these characteristics are beneficial for reducing the drying shrinkage of concrete.

### 3.4. Effects of Shrinkage Mitigation Methods on Internal Relative Humidity of Concrete

**Figure 10:**
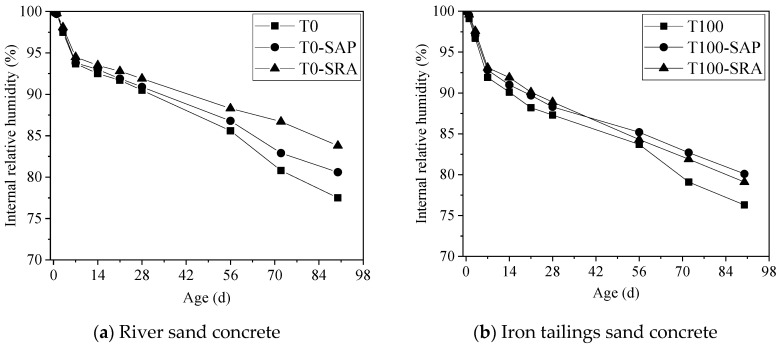
Development of internal RH of different concrete under autogenous shrinkage.

The development of internal RH is a result of dissolved salts in the pore solution and curvature of the fluid/vapour menisci [15,39]. Ions dissolved in the pore solution are responsible for the depression of water activity, which would lead to the decrease in internal RH [40,41]. Figure 10 presents influences of shrinkage mitigation methods on the development of the internal RH of river sand and iron tailings sand concrete under autogenous shrinkage. It should be mentioned that due to the limitation of test method, the drop of internal RH caused by ions dissolved in the pore fluid before setting could not be measured. As shown in Figure 10, the internal RH of all samples decreased rapidly before 7 d. Replacing river sand with iron tailings sand resulted in a lower internal RH, and hence higher tensile stresses developed in pores of iron tailings sand concrete, which led to a higher shrinkage. The faster internal RH reduction of iron tailings sand concrete could be attributed to the faster hydration of cement due to the presence of small calcite particles, as indicated in Section 3.2 of this paper. As the hydration of cement particles would increase the amounts of ions in pore solution and consume more water, the incorporating of SAP or the application of SRA could reduce the internal RH decrease. 

**Figure 11:**
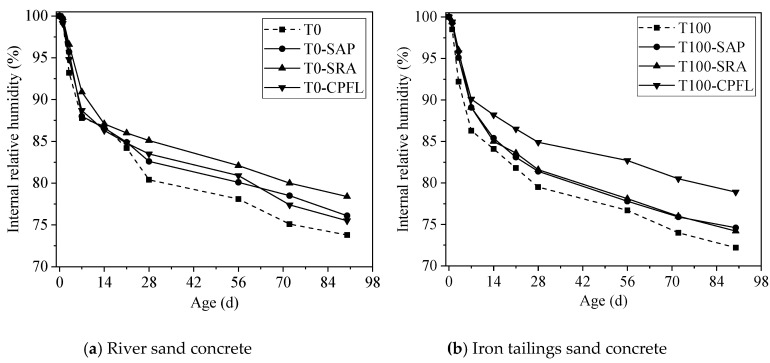
Development of internal RH of different concrete under drying shrinkage.

For the drying environment, apart from the water consumed by cement particles hydration, the water exchange with the environment would also cause moisture loss [42,43]. The development of an internal RH of the concretes under drying shrinkage is plotted in Figure 11, which also shows a similar variation trend with autogenous shrinkage. For river sand concrete, the internal RH of concrete-contained SRA was higher than concrete-applying SAP or CPFL, and all of these were higher than the control group. For iron tailings sand concrete, the use of SAP, SRA and CPFL could also increase the internal RH of concrete, albeit to a different extent. More specifically, the application of CPFL increased the internal RH of concrete remarkably, while lower effects of SRA and SAP on internal RH could be identified. Furthermore, no significant difference on internal RH could be observed before 14 days after applying shrinkage reducing approaches, while a much more obvious impact could be found after 3 d for iron tailings sand concrete, which is in line with the drying shrinkage data.

Since SAP was added in powder form, it could absorb water during concrete mixing. As the internal RH dropped, water was released from SAP’s reservoir, thus the internal RH decrease would be delayed. The SRA could also improve the internal RH by slowing down the evaporation rate of water. The application of CPFL was cable of enhancing the quality of concrete in the cover region and improve the permeability of concrete, thus reducing water loss and increasing the internal RH.

### 3.5. Effects of Shrinkage-Reducing Methods on Properties of Concrete

**Figure 12:**
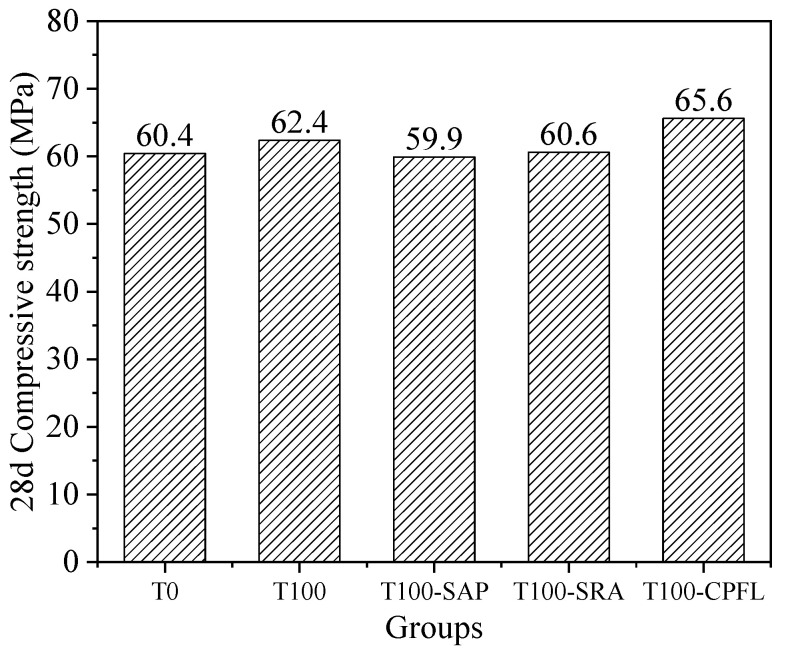
Effects of shrinkage-reducing methods on 28 d compressive strength of iron tailings sand concrete.

The impacts of shrinkage-reducing methods on 28 d compressive strength is presented in Figure 12. It is clear that the compressive strength of iron tailings sand concretes with the addition of SAP and SRA were comparable to that of the river sand and iron tailings sand concrete without applying shrinkage-reducing measures, while the application of CPFL slightly increased the compressive strength of iron tailings sand concrete. Studies with respect to those three shrinkage-reducing methods on later age compressive strength also showed that they did not have any significant influence on the strength of concrete [18,37,44]. 

**Figure 13:**
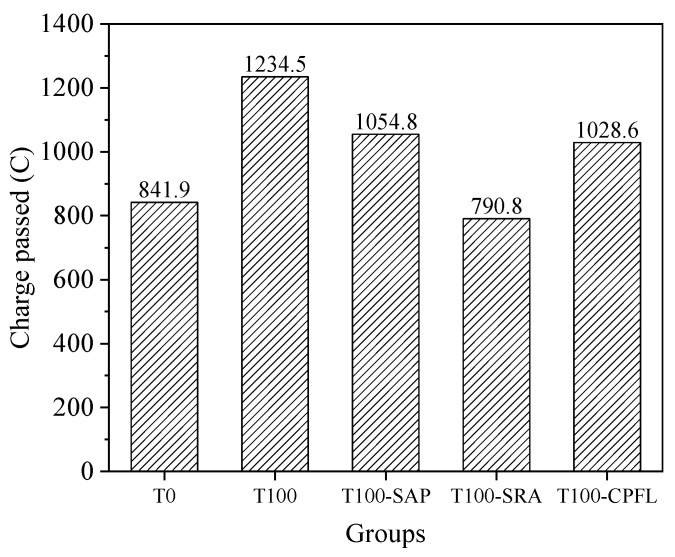
Resistance to chloride penetration of concrete with iron tailings sand and river sand.

Figure 13 shows the results of rapid chloride penetration tests for river sand and iron sand concrete with and without applying shrinkage mitigation methods. It is obvious that the substitution of river sand with iron tailings sand increased passed charge significantly, about 46.6% higher than the control group. The charges passed of river sand concrete were within the range of 0–1000 C, which are classified as very low chloride penetration according to ASTM C1202 standard, while the charges of iron tailings sand concrete were classified as low chloride penetration. The application of SAP, SRA and CPFL could reduce passed charges of iron tailings concrete by approximately 14.5%, 35.9% and 16.7%, respectively. It is reported that the extra water absorbed by SAP could increase the hydration degree of cement and improve the pore connectivity, which would result in a lower chloride migration coefficient [45]. Assmann [46] also investigated the impact of SAP on the chloride penetration resistance of normal and high-strength concrete, and found that SAP particles smaller than 125 μm had a positive effect on this performance, while SAP particles within 125–500 μm would act in the opposite role. There are two reasons for the decrease of passed charges in concrete containing SRA: the first is that the viscosity of pore solution increased and chloride diffusion was slowed down, since the flow rate of a fluid in concrete was strongly related to the viscosity of the fluid [47]; and the second is that a higher pore solution viscosity and lower surface tension would also reduce the sorptivity of concrete [47,48]. The CPFL could improve the chloride penetration resistance by densifying the surface of the concrete.

## 4. Conclusions

The sieving curves and fineness modulus of the iron tailings sand were similar with the river sand, and it can be utilized as a complete replacement of the river sand to produce concrete with comparable workability, and a higher mechanical strength and elasticity modulus. Meanwhile, the utilization of iron tailings sand can eliminate its negative effects on the environment, and protect the river channels through the preservation of the river sand. However, the shrinkage of iron tailings sand concrete was significantly larger than the river sand concrete, which may cause a high risk of cracking and shorten the service life of buildings. Therefore, it is necessary to study strategies which can mitigate the autogenous and drying shrinkage of iron tailings sand concretes. This study presents the results and analysis conducted to investigate the effects of the application of SAP, SAR and CPFL on shrinkage characteristics of the iron tailings sand concrete. From the results obtained, the following conclusions can be drawn:

1. The river sand was replaced by the iron tailings sand of the same mass in this study. Under this condition, the autogenous and total shrinkage of the iron tailings sand concrete were 9.8% and 13.3% higher than the river sand concrete at the age of 90 d, respectively, which could be ascribed to the accelerated hydration rate brought by the small calcite particles, lower volume ratio and poor water-retaining property of the iron tailings sand. 

2. Approximately 30% and 18% reduction in the autogenous shrinkage of iron tailings sand concrete was obtained at 90 days by incorporation of SAP and SRA, respectively. For total shrinkage of iron tailings sand concrete with SAP and SRA, a similar reduction (25% and 21%) was obtained at 90 days, while a much higher reduction (41%) was obtained using CPFL. Comparing shrinkage behaviors of the river sand and iron tailings sand concrete, it could be found that the SRA was the most efficient shrinkage-reducing method for the river sand concrete, while for the iron tailings sand concrete, SAP performed better on autogenous shrinkage and CPFL had the most prominent reducing effect on total shrinkage. Furthermore, the shrinkage mitigation strategies did not make any noticeable difference for the total shrinkage behavior of river sand concrete before 14 days, while far more obvious effects could be observed in iron tailings sand concrete after 3 days.

3. The application of SAP, SRA and CPFL was effective in reducing the autogenous and total shrinkage of iron tailings sand concrete, and they had no negative impacts on the mechanical property and chloride penetration resistance of concrete. Therefore, through implementing some measures, concretes with properties no worse than river sand concrete could be produced by using iron tailings sand.

## Figures and Tables

**Table 1:** Chemical composition of cement, ground granulated blast furnace slag (GGBFS) and fly ash (wt%).

Composition	SiO_2_	Al_2_O_3_	Fe_2_O_3_	K_2_O	CaO	MgO	TiO_2_	Na_2_O	SO_3_	LOI
PC	21.54	6.22	4.47	0.84	59.21	2.61	0.51	0.23	2.32	1.57
GGBFS	33.04	14.48	0.44	0.34	37.40	9.63	1.33	0.27	2.56	0.84
Fly ash	46.02	25.46	16.46	0.22	1.27	0.53	0.12	1.57	1.58	4.50

**Table 2:** Chemical composition of iron tailings sand (wt%).

Composition	SiO_2_	Al_2_O_3_	Fe_2_O_3_	K_2_O	CaO	MgO	TiO_2_	Na_2_O	SO_3_	P_2_O_5_	LOI
Content	36.94	10.38	16.96	0.37	11.04	7.94	5.97	1.19	0.22	3.38	4.53

**Table 3:** Properties of controlled permeable formwork liner.

Thickness/mm	Width/m	Fracture Strength	
		Longitudinal/kN·m^−1^	Transverse/kN·m^−1^
3	2	8.2	6.2

**Table 4:** Mix proportion of iron tailings sand concrete (kg/m^3^).

Group	Cement	Fly Ash	Slag	River Sand	Tailings Sand	Gravel	Water	Super-Plasticizer	SAP	SRA	Wa/B
T0	294	63	63	743.8	0	1093.7	147	4.2	-	-	-
T100	294	63	63	0	743.8	1093.7	147	4.2	-	-	-
T0-SAP	294	63	63	743.8	0	1093.7	147	4.2	2.52	-	0.05
T100-SAP	294	63	63	0	743.8	1093.7	147	4.2	2.52	-	0.05
T0-SRA	294	63	63	743.8	0	1093.7	147	4.2	-	4.2	-
T100-SRA	294	63	63	0	743.8	1093.7	147	4.2	-	4.2	-
T0-CPFL	294	63	63	743.8	0	1093.7	147	4.2	-	-	-
T100-CPFL	294	63	63	0	743.8	1093.7	147	4.2	-	-	-

**Table 5:** Properties of concrete with river sand and iron tailings sand.

Groups	Slump (mm)	Slump Flow (mm)	Compressive Strength (MPa)		Flexural Strength (MPa)		Modulus of Elasticity at 28 d (GPa)
			7 d	28 d	7 d	28 d	
T0	200	510	46.9 ± 1.8	60.4 ± 2.3	4.8 ± 0.3	8.0 ± 0.6	51.6 ± 3.1
T100	190	510	49.3 ± 2.0	62.4 ± 2.6	5.5 ± 0.3	8.5 ± 0.5	54.4 ± 3.4
